# Bis(acetonitrile-κ*N*)(1,10-phenanthroline-κ^2^ *N*,*N*′)platinum(II) bis­(perchlorate)

**DOI:** 10.1107/S1600536810008299

**Published:** 2010-03-10

**Authors:** Kwang Ha

**Affiliations:** a School of Applied Chemical Engineering, The Research Institute of Catalysis, Chonnam National University, Gwangju 500-757, Republic of Korea

## Abstract

The asymmetric unit of the title compound, [Pt(CH_3_CN)_2_(C_12_H_8_N_2_)](ClO_4_)_2_, contains one half of a cationic Pt^II^ complex and pair of half perchlorate anions, one of which is disordered over two sites in a 0.53 (3):0.47 (3) ratio. The complex and anions are disposed about a crystallographic mirror plane parallel to the ac plane passing through the Pt and Cl atoms. In the complex, the Pt^II^ ion lies in a distorted square-planar environment defined by four N atoms of the chelating 1,10-phenanthroline ligand and two distinct acetonitrile mol­ecules. The component ions inter­act by means of inter­molecular C—H⋯O hydrogen bonds.

## Related literature

For the synthesis of [PtCl_2_(phen)] (phen = 1,10-phenanthroline), see: Hodges & Rund (1975 ▶). For the crystal structure of [Pd(phen)(CH_3_CN)_2_](O_3_SCF_3_)_2_, see: Adrian *et al.* (2008 ▶).
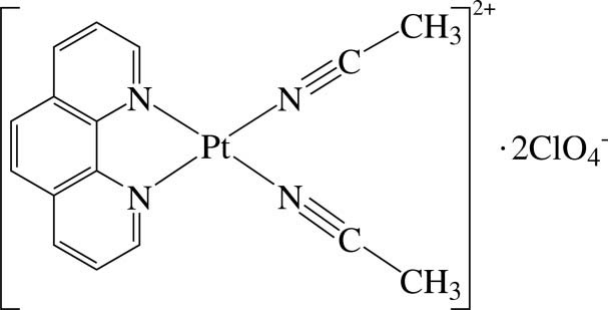


## Experimental

### 

#### Crystal data

[Pt(C_2_H_3_N)_2_(C_12_H_8_N_2_)](ClO_4_)_2_
*M* *_r_* = 656.30
Orthorhombic, 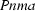

*a* = 9.1407 (5) Å
*b* = 11.7822 (7) Å
*c* = 18.3215 (11) Å
*V* = 1973.2 (2) Å^3^
*Z* = 4
Mo *K*α radiation
μ = 7.44 mm^−1^
*T* = 200 K
0.28 × 0.12 × 0.04 mm

#### Data collection

Bruker SMART 1000 CCD diffractometer
Absorption correction: multi-scan (*SADABS*; Bruker, 2000 ▶) *T*_min_ = 0.763, *T*_max_ = 1.000
11860 measured reflections
2043 independent reflections
1540 reflections with *I* > 2σ(*I*)
*R*_int_ = 0.087

#### Refinement

*R*[*F* ^2^ > 2σ(*F* ^2^)] = 0.042
*wR*(*F* ^2^) = 0.100
*S* = 1.02
2043 reflections
176 parameters
18 restraints
H-atom parameters constrained
Δρ_max_ = 2.23 e Å^−3^
Δρ_min_ = −1.82 e Å^−3^

### 

Data collection: *SMART* (Bruker, 2000 ▶); cell refinement: *SAINT* (Bruker, 2000 ▶); data reduction: *SAINT*; program(s) used to solve structure: *SHELXS97* (Sheldrick, 2008 ▶); program(s) used to refine structure: *SHELXL97* (Sheldrick, 2008 ▶); molecular graphics: *ORTEP-3* (Farrugia, 1997 ▶) and *PLATON* (Spek, 2009 ▶); software used to prepare material for publication: *SHELXL97*.

## Supplementary Material

Crystal structure: contains datablocks global, I. DOI: 10.1107/S1600536810008299/pk2230sup1.cif

Structure factors: contains datablocks I. DOI: 10.1107/S1600536810008299/pk2230Isup2.hkl

Additional supplementary materials:  crystallographic information; 3D view; checkCIF report

## Figures and Tables

**Table 1:** Selected bond angles (°)

N2^i^—Pt1—N2	87.9 (3)
N1—Pt1—N1^i^	81.9 (3)

Symmetry code: (i) 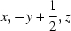.

**Table 2:** Hydrogen-bond geometry (Å, °)

*D*—H⋯*A*	*D*—H	H⋯*A*	*D*⋯*A*	*D*—H⋯*A*
C1—H1⋯O6^ii^	0.95	2.60	3.48 (2)	155
C3—H3⋯O2^iii^	0.95	2.54	3.210 (8)	127
C3—H3⋯O3^iv^	0.95	2.54	3.486 (11)	178
C7—H7*A*⋯O6^ii^	0.98	2.39	3.066 (18)	126
C7—H7*B*⋯O3^v^	0.98	2.41	3.143 (11)	131

Symmetry codes: (ii) 
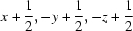
; (iii) 
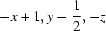
; (iv) 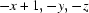; (v) 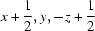.

**Fig. 1.**
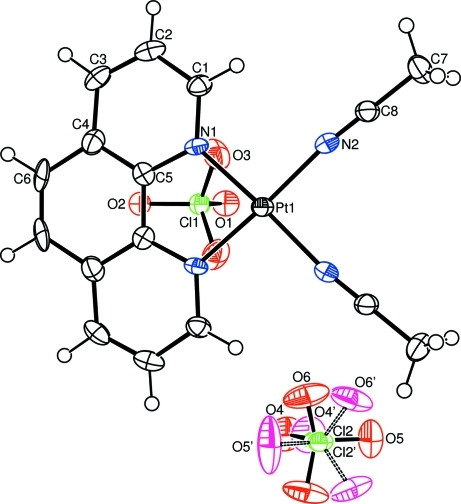
The structure of the title compound with displacement ellipsoids drawn at the 50% probability level for non-H atoms. Unlabelled atoms are related to labelled atoms by the symmetry operation and the bonds of the minor components of the disordered ClO4 anion are shown with dashed lines.

**Fig. 2.**
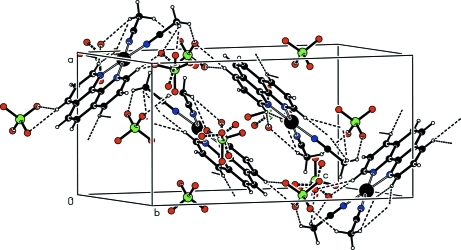
View of the unit-cell contents of the title compound. Hydrogen-bond interactions are drawn with dashed lines.

## supplementary crystallographic information

### Comment

The asymmetric unit of the title compound, [Pt(phen)(CH_3_CN)_2_](ClO_4_)_2_ (where phen is 1,10-phenanthroline, C_12_H_8_N_2_), contains one half of a cationic Pt^II^ complex and half a perchlorate anion (Fig. 1). The complex and anions are disposed about a crystallographic mirror plane parallel to the ac plane passing through the Pt and Cl atoms (Fig. 2). In the complex, the Pt^II^ ion lies in a distorted square-planar environment defined by four N atoms of the chelating 1,10-phenanthroline ligand and two distinct acetonitrile molecules. The main contribution to the distortion is the tight N1—Pt1—N1^i^ [symmetry code: (i) x,-y+1/2,z] chelate angle [81.9 (3)°], which results in non-linear trans arrangement [<N1—Pt1—N2^i^ = 177.0 (2)°]. The Pt—N bond lengths are almost equal [Pt1—N(phen): 2.001 (6) Å; Pt1—N(CH_3_CN): 1.994 (7) Å] (Table 1). The component ions interact by means of intermolecular C—H···O hydrogen bonds (Fig. 2 and Table 2).

### Experimental

To a solution of AgClO_4_.H_2_O (0.1006 g, 0.446 mmol) in CH_3_CN (70 ml) was added [PtCl_2_(phen)] (0.0996 g, 0.223 mmol) and refluxed for 7 h. The mixture was filtered to remove AgCl and then the resulting solution was dried under vacuum. The residue was washed with CH_2_Cl_2_ and dried at 50 °C, to give a pale gray powder (0.1401 g). Crystals suitable for X-ray analysis were obtained by slow evaporation from a CH_3_CN solution.

### Refinement

H atoms were positioned geometrically and allowed to ride on their respective parent atoms [C—H = 0.95 (aromatic) or 0.98 Å (CH_3_) and U_iso_(H) = 1.2U_eq_(C) or 1.5U_eq_(methyl C)]. The highest peak (2.23 e Å^-3^) and the deepest hole (-1.82 e Å^-3^) in the difference Fourier map are located 0.86 and 0.96 Å, respectively, from the atom Pt1. The O atoms (O4, O5 and O6) of the ClO_4_ anion displayed relatively large displacement factors so that the anion appears to be partially disordered. The anion was modelled as disordered over two sites with a major site occupancy factor of 0.53 (3). A total of 18 restraints were used in the refinement using the following SHELXL97 (Sheldrick, 2008) commands: SAME 0.020 Cl2' > O6' and DELU 0.010 Cl2 > O6'.  In addition, the displacement parameters of the major and minor component atoms Cl2 Cl2' were constrained using the EADP command.

### Crystal data

**Table:**

[Pt(C_2_H_3_N)_2_(C_12_H_8_N_2_)](ClO_4_)_2_	*F*(000) = 1256
*M**_r_* = 656.30	*D*_x_ = 2.209 Mg m^−^^3^
Orthorhombic, *P**n**m**a*	Mo *K*α radiation, λ = 0.71073 Å
Hall symbol: -P 2ac 2n	Cell parameters from 3403 reflections
*a* = 9.1407 (5) Å	θ = 2.2–25.0°
*b* = 11.7822 (7) Å	µ = 7.44 mm^−^^1^
*c* = 18.3215 (11) Å	*T* = 200 K
*V* = 1973.2 (2) Å^3^	Rod, colorless
*Z* = 4	0.28 × 0.12 × 0.04 mm

### Data collection

**Table:**

Bruker SMART 1000 CCD diffractometer	2043 independent reflections
Radiation source: fine-focus sealed tube	1540 reflections with *I* > 2σ(*I*)
graphite	*R*_int_ = 0.087
φ and ω scans	θ_max_ = 26.0°, θ_min_ = 2.1°
Absorption correction: multi-scan (*SADABS*; Bruker, 2000)	*h* = −11→7
*T*_min_ = 0.763, *T*_max_ = 1.000	*k* = −14→14
11860 measured reflections	*l* = −22→21

### Refinement

**Table:**

Refinement on *F*^2^	Primary atom site location: structure-invariant direct methods
Least-squares matrix: full	Secondary atom site location: difference Fourier map
*R*[*F*^2^ > 2σ(*F*^2^)] = 0.042	Hydrogen site location: inferred from neighbouring sites
*wR*(*F*^2^) = 0.100	H-atom parameters constrained
*S* = 1.02	*w* = 1/[σ^2^(*F*_o_^2^) + (0.0514*P*)^2^] where *P* = (*F*_o_^2^ + 2*F*_c_^2^)/3
2043 reflections	(Δ/σ)_max_ < 0.001
176 parameters	Δρ_max_ = 2.23 e Å^−^^3^
18 restraints	Δρ_min_ = −1.82 e Å^−^^3^

### Special details

**Table:**

Geometry. All esds (except the esd in the dihedral angle between two l.s. planes) are estimated using the full covariance matrix. The cell esds are taken into account individually in the estimation of esds in distances, angles and torsion angles; correlations between esds in cell parameters are only used when they are defined by crystal symmetry. An approximate (isotropic) treatment of cell esds is used for estimating esds involving l.s. planes.
Refinement. Refinement of *F*^2^ against ALL reflections. The weighted *R*-factor wR and goodness of fit *S* are based on *F*^2^, conventional *R*-factors *R* are based on *F*, with *F* set to zero for negative *F*^2^. The threshold expression of *F*^2^ > σ(*F*^2^) is used only for calculating *R*-factors(gt) etc. and is not relevant to the choice of reflections for refinement. *R*-factors based on *F*^2^ are statistically about twice as large as those based on *F*, and *R*- factors based on ALL data will be even larger.

### Fractional atomic coordinates and isotropic or equivalent isotropic displacement parameters (Å^2^)

**Table:**

	*x*	*y*	*z*	*U*_iso_*/*U*_eq_	Occ. (<1)
Pt1	0.96551 (5)	0.2500	0.10603 (2)	0.02158 (17)	
N1	0.8473 (6)	0.1387 (5)	0.0483 (3)	0.0219 (14)	
N2	1.0774 (7)	0.1326 (5)	0.1611 (4)	0.0278 (16)	
C1	0.8490 (8)	0.0253 (6)	0.0516 (4)	0.0264 (18)	
H1	0.9141	−0.0111	0.0845	0.032*	
C2	0.7580 (9)	−0.0405 (7)	0.0081 (5)	0.034 (2)	
H2	0.7628	−0.1209	0.0110	0.041*	
C3	0.6622 (9)	0.0097 (7)	−0.0387 (5)	0.031 (2)	
H3	0.5999	−0.0355	−0.0682	0.038*	
C4	0.6557 (8)	0.1302 (7)	−0.0431 (5)	0.0304 (19)	
C5	0.7512 (7)	0.1902 (6)	0.0018 (4)	0.0229 (17)	
C6	0.5587 (9)	0.1932 (8)	−0.0901 (4)	0.034 (2)	
H6	0.4937	0.1535	−0.1215	0.041*	
C7	1.2397 (10)	0.0000 (8)	0.2375 (5)	0.040 (2)	
H7A	1.2170	−0.0791	0.2254	0.061*	
H7B	1.2217	0.0129	0.2896	0.061*	
H7C	1.3427	0.0154	0.2264	0.061*	
C8	1.1474 (8)	0.0750 (6)	0.1947 (4)	0.0255 (18)	
Cl1	0.4808 (3)	0.2500	0.14861 (16)	0.0273 (6)	
O1	0.3890 (9)	0.2500	0.2122 (5)	0.040 (2)	
O2	0.3939 (10)	0.2500	0.0837 (4)	0.040 (2)	
O3	0.5699 (7)	0.1506 (6)	0.1490 (4)	0.0515 (19)	
Cl2	0.430 (2)	0.7500	0.341 (4)	0.033 (2)	0.53 (3)
O4	0.272 (2)	0.7500	0.340 (2)	0.080 (10)	0.53 (3)
O5	0.468 (3)	0.7500	0.4176 (11)	0.088 (10)	0.53 (3)
O6	0.4825 (19)	0.6495 (12)	0.3106 (12)	0.077 (8)	0.53 (3)
Cl2'	0.461 (3)	0.7500	0.340 (4)	0.033 (2)	0.47 (3)
O4'	0.312 (3)	0.7500	0.365 (2)	0.068 (10)	0.47 (3)
O5'	0.453 (3)	0.7500	0.2625 (11)	0.111 (15)	0.47 (3)
O6'	0.5367 (19)	0.6564 (15)	0.3651 (15)	0.073 (8)	0.47 (3)

### Atomic displacement parameters (Å^2^)

**Table:**

	*U*^11^	*U*^22^	*U*^33^	*U*^12^	*U*^13^	*U*^23^
Pt1	0.0226 (3)	0.0172 (2)	0.0250 (3)	0.000	−0.00007 (19)	0.000
N1	0.027 (4)	0.014 (3)	0.025 (4)	−0.002 (2)	0.003 (3)	−0.003 (3)
N2	0.032 (4)	0.020 (3)	0.031 (4)	−0.005 (3)	−0.005 (3)	−0.003 (3)
C1	0.026 (4)	0.022 (4)	0.031 (5)	0.004 (3)	0.001 (3)	−0.005 (3)
C2	0.038 (5)	0.020 (4)	0.044 (6)	−0.006 (4)	0.006 (4)	−0.006 (4)
C3	0.031 (5)	0.030 (5)	0.034 (5)	−0.012 (4)	0.003 (4)	−0.010 (4)
C4	0.028 (5)	0.033 (5)	0.030 (5)	0.001 (3)	−0.004 (4)	−0.006 (4)
C5	0.019 (4)	0.027 (4)	0.022 (4)	0.000 (3)	0.003 (3)	0.002 (3)
C6	0.037 (5)	0.057 (6)	0.008 (4)	−0.011 (4)	0.000 (3)	−0.005 (3)
C7	0.047 (6)	0.038 (5)	0.036 (6)	0.012 (4)	−0.012 (4)	0.002 (4)
C8	0.025 (4)	0.020 (4)	0.032 (5)	−0.001 (3)	−0.002 (4)	0.000 (4)
Cl1	0.0272 (14)	0.0256 (14)	0.0292 (17)	0.000	−0.0033 (12)	0.000
O1	0.038 (5)	0.039 (5)	0.042 (6)	0.000	0.004 (4)	0.000
O2	0.055 (6)	0.031 (5)	0.035 (5)	0.000	−0.014 (4)	0.000
O3	0.058 (4)	0.055 (4)	0.041 (4)	0.033 (3)	−0.003 (3)	−0.003 (3)
Cl2	0.030 (7)	0.0226 (15)	0.047 (3)	0.000	−0.003 (11)	0.000
O4	0.031 (9)	0.077 (18)	0.13 (3)	0.000	−0.017 (11)	0.000
O5	0.11 (2)	0.09 (2)	0.063 (10)	0.000	−0.040 (13)	0.000
O6	0.072 (11)	0.034 (8)	0.124 (19)	0.002 (8)	0.025 (12)	−0.031 (10)
Cl2'	0.030 (7)	0.0226 (15)	0.047 (3)	0.000	−0.003 (11)	0.000
O4'	0.023 (11)	0.09 (2)	0.09 (2)	0.000	0.005 (13)	0.000
O5'	0.09 (2)	0.21 (4)	0.034 (9)	0.000	−0.016 (11)	0.000
O6'	0.057 (11)	0.037 (10)	0.12 (2)	0.013 (8)	0.007 (11)	0.028 (12)

### Geometric parameters (Å, °)

**Table:**

Pt1—N2^i^	1.994 (7)	C6—H6	0.9500
Pt1—N2	1.994 (7)	C7—C8	1.452 (11)
Pt1—N1	2.001 (6)	C7—H7A	0.9800
Pt1—N1^i^	2.001 (6)	C7—H7B	0.9800
N1—C1	1.337 (9)	C7—H7C	0.9800
N1—C5	1.366 (9)	Cl1—O3^i^	1.427 (6)
N2—C8	1.118 (9)	Cl1—O3	1.427 (6)
C1—C2	1.388 (11)	Cl1—O2	1.430 (9)
C1—H1	0.9500	Cl1—O1	1.436 (9)
C2—C3	1.360 (12)	Cl2—O6^ii^	1.40 (3)
C2—H2	0.9500	Cl2—O6	1.40 (3)
C3—C4	1.424 (11)	Cl2—O5	1.44 (7)
C3—H3	0.9500	Cl2—O4	1.44 (2)
C4—C5	1.392 (10)	Cl2'—O6'^ii^	1.38 (3)
C4—C6	1.442 (11)	Cl2'—O6'	1.38 (3)
C5—C5^i^	1.410 (14)	Cl2'—O5'	1.42 (8)
C6—C6^i^	1.338 (19)	Cl2'—O4'	1.44 (2)
			
N2^i^—Pt1—N2	87.9 (3)	C4—C6—H6	119.5
N2^i^—Pt1—N1	177.0 (2)	C8—C7—H7A	109.5
N2—Pt1—N1	95.1 (2)	C8—C7—H7B	109.5
N2^i^—Pt1—N1^i^	95.1 (2)	H7A—C7—H7B	109.5
N2—Pt1—N1^i^	177.0 (2)	C8—C7—H7C	109.5
N1—Pt1—N1^i^	81.9 (3)	H7A—C7—H7C	109.5
C1—N1—C5	118.6 (6)	H7B—C7—H7C	109.5
C1—N1—Pt1	128.7 (5)	N2—C8—C7	179.2 (9)
C5—N1—Pt1	112.7 (5)	O3^i^—Cl1—O3	110.4 (6)
C8—N2—Pt1	173.4 (6)	O3^i^—Cl1—O2	108.7 (3)
N1—C1—C2	121.7 (7)	O3—Cl1—O2	108.7 (3)
N1—C1—H1	119.1	O3^i^—Cl1—O1	109.3 (3)
C2—C1—H1	119.1	O3—Cl1—O1	109.3 (3)
C3—C2—C1	120.3 (8)	O2—Cl1—O1	110.5 (6)
C3—C2—H2	119.9	O6^ii^—Cl2—O6	116 (4)
C1—C2—H2	119.9	O6^ii^—Cl2—O5	108 (3)
C2—C3—C4	119.7 (7)	O6—Cl2—O5	108 (3)
C2—C3—H3	120.1	O6^ii^—Cl2—O4	110 (2)
C4—C3—H3	120.1	O6—Cl2—O4	110 (2)
C5—C4—C3	116.5 (7)	O5—Cl2—O4	105 (3)
C5—C4—C6	118.5 (7)	O6'^ii^—Cl2'—O6'	106 (4)
C3—C4—C6	125.0 (8)	O6'^ii^—Cl2'—O5'	111 (3)
N1—C5—C4	123.1 (7)	O6'—Cl2'—O5'	111 (3)
N1—C5—C5^i^	116.3 (4)	O6'^ii^—Cl2'—O4'	111 (3)
C4—C5—C5^i^	120.5 (5)	O6'—Cl2'—O4'	111 (3)
C6^i^—C6—C4	121.0 (5)	O5'—Cl2'—O4'	106 (4)
C6^i^—C6—H6	119.5		
			
N2—Pt1—N1—C1	1.5 (7)	C1—N1—C5—C4	−0.7 (11)
N1^i^—Pt1—N1—C1	−178.3 (5)	Pt1—N1—C5—C4	−179.0 (6)
N2—Pt1—N1—C5	179.6 (5)	C1—N1—C5—C5^i^	178.5 (5)
N1^i^—Pt1—N1—C5	−0.2 (6)	Pt1—N1—C5—C5^i^	0.2 (5)
C5—N1—C1—C2	1.2 (11)	C3—C4—C5—N1	−0.1 (11)
Pt1—N1—C1—C2	179.2 (6)	C6—C4—C5—N1	179.9 (7)
N1—C1—C2—C3	−1.0 (12)	C3—C4—C5—C5^i^	−179.2 (5)
C1—C2—C3—C4	0.2 (12)	C6—C4—C5—C5^i^	0.8 (9)
C2—C3—C4—C5	0.3 (12)	C5—C4—C6—C6^i^	−0.8 (9)
C2—C3—C4—C6	−179.7 (8)	C3—C4—C6—C6^i^	179.2 (6)

Symmetry codes: (i) *x*, −*y*+1/2, *z*; (ii) *x*, −*y*+3/2, *z*.

### Hydrogen-bond geometry (Å, °)

**Table:**

*D*—H···*A*	*D*—H	H···*A*	*D*···*A*	*D*—H···*A*
C1—H1···O6^iii^	0.95	2.60	3.48 (2)	155
C3—H3···O2^iv^	0.95	2.54	3.210 (8)	127
C3—H3···O3^v^	0.95	2.54	3.486 (11)	178
C7—H7A···O6^iii^	0.98	2.39	3.066 (18)	126
C7—H7B···O3^vi^	0.98	2.41	3.143 (11)	131

Symmetry codes: (iii) *x*+1/2, −*y*+1/2, −*z*+1/2; (iv) −*x*+1, *y*−1/2, −*z*; (v) −*x*+1, −*y*, −*z*; (vi) *x*+1/2, *y*, −*z*+1/2.
